# Literature Review on the Pre-Slaughter Welfare of Italian Heavy Pigs

**DOI:** 10.3390/ani11123352

**Published:** 2021-11-24

**Authors:** Marika Vitali, Luca Sardi, Giovanna Martelli, Eleonora Nannoni

**Affiliations:** Department of Veterinary Medical Sciences, University of Bologna, 40064 Ozzano Emilia, Italy; marika.vitali4@unibo.it (M.V.); giovanna.martelli@unibo.it (G.M.); eleonora.nannoni2@unibo.it (E.N.)

**Keywords:** animal welfare, swine, heavy pigs, typical products, transport, slaughter, stunning

## Abstract

**Simple Summary:**

Italian heavy pigs production differs from pig farming in other countries of the world mainly due to the high body weight and age at slaughter of the animals. This implies that peculiar animal needs may be addressed to achieve a high level of animal welfare during the pre-slaughter phases. This narrative review aims to collect the available information on welfare issues of Italian heavy pigs in the pre-slaughter phases, and to highlight recent findings and knowledge gaps.

**Abstract:**

This work provides a narrative review of the available information on the welfare of Italian heavy pigs in the pre-slaughter phase (transport, lairage, and stunning). The meat from these pigs is used for specific PDO (Protected Designation of Origin) products, and the production rules for these specialties require higher body weight (160–170 kg) and age (in general more than 9 months) at slaughter than in most other countries. This may lead to specific behavioral and physiological needs of pigs. The present paper summarizes the main research findings and knowledge gaps for each of the pre-slaughter phases. Studies are presented according to the four principles of the Welfare Quality assessment protocol (good feeding, good housing, good health, and appropriate behavior). The results of the literature review indicate a lack of knowledge on several aspects. Most of studies were carried out in a single slaughterhouse, making it difficult to identify risk factors and confounding effects. Moreover, animal-based measures were assessed using different protocols, reducing the possibility of comparison across studies. These findings may serve as a basis for the development of specific research studies and policies aimed at enhancing the animal welfare level and the ethical attributes of this renowned production, also in accordance with consumers’ expectations.

## 1. Introduction

Consumers’ concern about the welfare of farmed animals has increased in recent years, progressively including implicit quality attributes such as the ethical value of the animal-derived products they purchase [1,2]. This is particularly relevant in the case of Protected Designation of Origin (PDO) products. A large proportion of consumers, in fact, expect these specialties to have been produced according to high/very high animal welfare standards [2]. Moreover, consumers expressed many concerns about slaughtering procedures [3,4].

Recently, an extensive report from EFSA (European Food Safety Authority) reviewed the risk factors determining poor welfare for pigs at slaughter [5]. The report highlighted the need to identify more indicators, especially animal-based measures (ABMs) and to validate them.

If some of the pre-slaughter hazards are common to any type of pig, others are peculiar of Italian heavy pigs intended for PDO products. These pigs, whose production rules are regulated by Consortia (the most known of which is the Parma Ham Consortium [6]), differ from pigs raised in most other countries for their age (more than 9 months) and body weight (approximatively 160–170 kg) at slaughter. Moreover, these pigs must belong only to specific genotypes (e.g., Italian Large White, Italian Landrace, Italian Duroc crossbreeds, and hybrids deriving from authorized cross-breeding programs) [6]. The higher age at slaughter implies that animals can reach sexual maturity. Males, therefore, need to be castrated to preserve meat quality and reduce aggression and unwanted mating [7]. Additionally, the production rules set the parameters for the quality of the raw thighs (the so-called “green hams”) and the meat processing techniques (preparation and curing of the thighs, qualitative parameters of the final product), and guarantee the traceability along the whole production chain [6].

Overall, the peculiarities of these pigs (mainly related to their high body weight and rearing technologies) may impact on their physiology and welfare needs [8]. The aim of this paper is to review the available literature on the welfare of Italian heavy pigs in the pre-slaughter phase and how it could be monitored using ABMs. Achieving higher levels of animal welfare in the pre-slaughtering phases would improve the intrinsic quality (enhanced ethical attributes) of the derived products, meeting consumers expectations.

Additionally, other countries worldwide have increased pigs’ slaughtering body weight over the past decades to dilute fixed costs [9]. In this scenario, studies on the welfare level of Italian heavy pigs could be beneficial also under other production contexts.

## 2. Methods

This manuscript narratively reviews studies focused on the welfare of Italian heavy pigs in the pre-slaughter phases (transport, lairage, and stunning). The literature search was conducted by consulting the databases ‘Web of Science’, ‘PubMed’, and ‘Scopus’. The search keywords initially used were “welfare”, “heavy pigs”, “transport”, “lairage”, “stunning”, and “slaughter”. Fifty-seven papers were obtained on Scopus, 14 on PubMed, and 9 on Web of Science. Duplicate papers and papers focusing solely on animal welfare on farm were removed. When necessary, additional searches were carried out on specific aspects, using also the reference list from the selected papers to identify possible additional references to be included in the present work. Papers were included when they referred to the Italian commercial reality and when they expressly referred to “heavy pigs” or “Italian heavy pigs”, as defined in the production rules of the main dry-cured products consortia [2]. As a result of this process, a total of 16 papers were retained and discussed. It is essential to highlight that, due to the minimal number of papers retrieved, we decided to provide a narrative review, as comprehensive as possible, of the limited number of studies found.

The collected studies are presented according to each pre-slaughter phase, namely: transport (including also loading and unloading), lairage, and stunning (including also lairage-to-stunning transfer, and restraint during stunning). This structure reflects the categorization proposed by the European Food Safety Authority (EFSA) [5]. The main findings are reported based on the principles detailed in the Welfare Quality^®^ protocol [10], applied to the specific pre-slaughter phase. The principles are: good feeding (absence of thirst and hunger), good housing (thermal comfort and environment), good health (injuries, absence of pain caused by management procedures), and appropriate behavior (absence of pain and fear, and good human–animal relationships). Whenever possible, the pre-slaughter factors have been associated with ABMs and then related to welfare consequences for the pigs. If lack of evidence or weak associations were observed, this is reported and discussed.

## 3. Results of the Literature Search

Of the retrieved papers (and since some papers dealt with more than one pre-slaughter phase), 10 papers concerned transport, 11 lairage, and 2 stunning (Table 1).

ABMs have been reported based on when they were measured: ante-mortem, post-mortem, or in both phases (Table 1). The different time of assessment is essential since the same ABM can lead to different results when measured ante- or post-mortem.

When comparing different studies, it should also be considered that the use of different protocols, measuring instruments, scoring systems, or statistical methods may affect the outcome. When possible, these differences have been reported and discussed throughout the review.

## 4. Transport (Including Loading and Unloading)

Transport is regulated by Council Regulation (EC) No 1/2005 of 22 December 2004 [28] on the protection of animals during transport and related operations.

Heavy pigs intended for PDO products shall be reared and slaughtered in a specific geographic area defined by the production rules of each Consortium. Therefore, very long transportation (e.g., more than 24 h or by plane or ship) does not apply to this type of production. However, an unforeseen extension of the travel journey could occur due to traffic conditions, accidents, or vehicle issues. Based on our literature search, only a few studies addressed the welfare conditions of Italian heavy pigs during transport.

### 4.1. Good Feeding during Transport

The legislation requires that during transportation pigs must have fresh water always available [28]. To ensure this, pigs also need to have adequate space to move and reach the drinkers.

With respect to hunger, Parma Ham Consortium requires that pigs shall be healthy, rested, and fasting before slaughter [29]. However, it is unclear whether and for how long this pre-slaughter fasting is extended under commercial conditions in Italian heavy pigs. In general, fasting before slaughter is beneficial to pig welfare and aims at avoiding transport sickness and hyperthermia [5]. However, according to the same report [5], long fasting (over 18 h) might worsen pig handling, leading to more irregular behaviors (e.g., pigs going backwards, round-turning, and vocalizing).

To the best of the authors’ knowledge, however, no official report provides information on the watering and fasting duration of Italian heavy pigs in the pre-slaughter phase.

### 4.2. Good Housing during Transport

According to the EU regulation [28], the loading density for pigs weighing around 100 kg should not exceed 235 kg/m^2^. Similarly, the “guide to good practices for the transport of pigs” [30], states that animals over 120 kg of body weight should be guaranteed a minimum floor space of 0.70 m^2^ in the truck. The welfare consequences of overcrowding in the truck include injuries, fatigue due to insufficient space to rest, dehydration due to difficulty to reach the drinkers, and heat stress under hot temperatures [30]. A few studies were found on this topic for heavy pigs, and only one [14] reported that the mean space allowance on trucks transporting Italian heavy pigs (0.74 m^2^/160 kg) was similar to data collected in other countries (0.51 m^2^/110 kg). According to these data, heavy pigs had the mandatory minimum space allowance on the truck, but this result needs to be further and constantly monitored.

In Italy, the temperature can easily exceed 30 °C during the summer months, with high relative humidity. This suggests that special care should be taken to avoid losses due to heat stress. Indeed, Nannoni et al. [18] observed that during summer the number of trucks having at least one dead animal during transport was 1.32 times more frequent compared to the winter season. The study was retrospectively conducted over 57 months and on a total of 3,344,730 pigs processed in a single slaughterhouse. High environmental temperatures during transport have been also associated with low meat quality in heavy pigs by Sardi et al. [21]. It is essential to consider that pigs have poor thermoregulation abilities through their skin, and temperatures above 20 °C are already challenging for their ability to cope [31]. Therefore, preventing losses and poor meat quality due to heat stress requires additional efforts (such as providing sufficient and accessible water, and avoiding travelling during the hottest hours of the day) and adequate ventilation and cooling systems (fans, sprinkling/misting devices) [30].

Moreover, position within the truck might affect pig welfare and stress. For example, a pilot study on deck levels observed higher norepinephrine levels in animals from the third deck [16], probably due to higher stress and muscular fatigue during loading and transport. Accordingly, the research carried out by Arduini et al. [11] showed higher Temperature Humidity Index (THI) in the middle and upper decks.

### 4.3. Good Health during Transport

According to the legislation, no animal shall be transported unless it is fit for the intended journey [28]. Transport is a stressor that can lead to the impairment of health conditions, and investigating the risk factors would allow better transport procedures with the aim to minimize stress [32]. Only a few studies are available on the health conditions of heavy pigs during transport.

Nanni Costa et al. [12] assessed skin blemishes and meat traits in Italian heavy pigs loaded with two systems (ramp vs. lift) and transported at two stocking densities (0.4 vs. 0.6 m^2^/100 kg of live weight). The assessment was carried out post-mortem. Results showed no significant effect of the treatment on ABMs and meat traits. Another study analyzing data from five countries reported that, compared to conventional pigs slaughtered in other countries, at unloading heavy pigs transported in Italy showed a lower percentage of sickness, panting, and DOAs (Dead On Arrival) (0.01%, 0.03%, and 0.00%, respectively) [14]. These results are encouraging and demonstrate that it is possible to reduce the percentage of sick animals at arrival until close to 0% [14]. The study hypothesizes that these differences could be due to the higher breeding standards under the Parma Ham PDO production scheme. Another possible explanation is that, compared to other countries, Italian heavy pigs tend to have relatively short travel durations [18,19,21]. Similarly, another study [25] reported only one death at unloading on more than 10,000 heavy pigs inspected over 6 months in one Italian abattoir. However, before confirming the hypothesis that pigs in this certification production scheme benefit from lower mortality, this data should be systematically compared with those obtained from the same slaughterhouse on non-PDO pigs of the same age and body weight, and this information is hard to find as non-certified pigs are in the vast majority of cases slaughtered at a younger age and lighter bodyweight. It is also worth noting that no specific requirements on animal protection during the transportation phase are detailed by the production rules.

Regarding DOA pigs (i.e., animals found dead after transportation, at unloading), pre-slaughter losses were found to be more likely both during summer, and if the journey lasted more than 90 min [18]. Accordingly, another study observed more DOAs in the summer months, with a peak in July (odds ratio: 1.22) and if the journey lasted more than two hours [19]. The same study also evidenced a significant increase of DOAs when high THIs (Temperature-Humidity Indexes) were recorded, with 78.5 THI being the threshold above which DOAs increased (corresponding to 35 °C and 80% of RH (Relative Humidity), or 30 °C and 50% RH, or 27 °C and 80% RH).

Increased THIs (average value of 65.5 ± 2.0) during short journeys (30 min) were also found to increase the lesion score on the quarters and body surface in 10 batches of pigs transported from the same farm [11]. The location of pigs within the truck did not affect skin lesions. The study hypothesized that hot conditions during the phases of transport resulted in more active pigs, prone to hit against loading and unloading facilities.

Some studies measured ham defects as a consequence of transport conditions. Some of these defects have been proposed as ABMs to assess the pre-slaughter conditions in heavy pigs, since they reflect injuries that occurred during pre-slaughter, for example hematomas, bone ruptures and muscle tears [24,25]. Arduini et al. [15] reported a positive correlation between transportation length (i.e., distance) and ham defects. Over two years, the study inspected more than 900,000 tights in a single abattoir, and evaluated hematomas, lacerations, micro-hemorrhages, and veining defects. Results showed that, when traveling distances were up to 170 km, all the ham defects decreased, while above 170 km they increased. An interaction between season and distance of transport was also observed. Overall, ham defects were lower during summer, with the lowest prevalence in tights deriving from pigs transported for 50–170 km during this season. According to the authors, the low prevalence of ham defects in summer could be an indirect effect of the higher care of the stockman when handling the animals, to reduce the risk of animal losses. However, this aspect has not been further investigated. Hematomas increased drastically during winter and autumn, especially when pigs were transported for more than 170 km [15].

Journey duration also affects stress-related hormones: cortisol and norepinephrine resulted to be progressively higher when comparing 3, 6.5, and 11 h of travelling [16]. Similarly, lower meat quality was observed at the sensory analysis in heavy pigs subjected to longer journeys and travel distances [21], confirming a negative effect on meat quality of longer journeys even when the travel duration was relatively short (average journey duration: 37.2 min, range 18–90; average distance traveled: 26.2 min, range 11–59). In particular, the authors observed lower meat color and higher final residue at the sensory analysis in animals that were more affected by pre-slaughter stressors in the transport phase.

With respect to road conditions, the study conducted by Sardi et al. [17] on 440 shipments of pigs (10 pigs/shipment on which a blood sample was collected at exsanguination) slaughtered all at the same plant, identified two clusters of pigs based on their level of blood cortisol and CK (Creatin Kinase): low-stress (LS, having lower cortisol and CK levels) and high-stress (HS, having higher cortisol and CK levels). Even if the study assessed an extensive range of pre-slaughter parameters, the variables most associated with the two clusters were transport distance and speed of travel. Surprisingly, LS pigs had undergone longer journey and higher speed, as compared to HS. According to the authors, considering that the average journey duration was short (below 40 min), it is likely that transports for the LS cluster were carried out on smoother roads (e.g., highways) instead of on rougher roads (e.g., country or city roads). This might explain the difference found, posing a question on road conditions in addition to transport distance or journey duration. Rougher travels, despite being shorter in duration, could have caused a higher stress response. The authors also hypothesized that animals may not have had time to recover from the stress experienced at loading during these very short travels.

### 4.4. Appropriate Behavior during Transport

Maintaining stable groups at loading, by avoiding mixing pigs from different pens, is considered a good practice. It prevents aggressive behaviors, and, in turn, reduces the risk of skin lesions and muscle-skeletal injuries. Italian heavy pigs showed lower skin lesions (measured on the carcasses) than lighter pigs from other countries [14]. The authors hypothesized that, because the majority of Italian pigs are raised for PDO production, which is subjected to high traceability requirements, this may imply an indirect reduction in the mixing of pigs throughout the production cycle, with a decrease in aggressions and subsequent lesions. However, this hypothesis should be confirmed by comparing these data with those from similar pigs non-adhering to a PDO scheme.

One of the welfare issues for all pigs during transport is the occurrence of fatigue, whose prevalence is largely unknown in Italian heavy pigs, as well as the knowledge about the occurrence of abnormal behaviors/accidents (e.g., slips, falls, overlaps) at loading and unloading. Behavioral studies using videotaping are recommended to assess the frequencies of abnormal behavior in the truck (e.g., mounting, trampling, or fighting), and to highlight the occurrence of behaviors indicating heat stress (e.g., panting), cold (e.g., huddling), or fatigue (e.g., protracted lying or sternal recumbency).

It is also important that stockpeople and abattoir personnel handle the animals with care, avoiding unnecessary fear and pain. One of the major risks in this sense is represented by moving the animals at loading and unloading. The legislation [28] states that the use of electric goads should be avoided as much as possible. Data on the use and consequences of these moving devices and on the prevalence of abnormal and dangerous behavior (running, slipping, falling, turning, reluctance to move), negative affective states (fear, pain, distress), physiological consequences (fatigue, muscle damage), and possible consequent traumatic injuries have not been studied in this population of pigs. The study by Calà et al. [20] measured behavior at loading and unloading in pigs from the same farm and transported to three different slaughterhouses. Rubber pipes were used to move the pigs both at loading and at unloading. Behavior was similar across groups at loading, while higher vocalization was recorded in one of the slaughterhouses, as a likely consequence of the increased use of rubber pipes to move the animals at unloading observed in the same slaughterhouse. These results highlight the importance of gentle handling procedures. In fact, vocalizations during handling have been associated with higher stress and rough handling procedures [33].

A recent study [21] reported that abnormal behaviors at unloading were associated with poor meat quality, showing increased tenderness and instrumental color alteration (redness and yellowness), that seems relatable to red-pink, soft, and exudative meat, defined as a mild occurrence of the PSE (Pale, Soft, and Exudative) defect [34]. These results highlight the importance of this aspect also from a productive point of view. Similarly, another study [17] identified that pigs showing more irregular behaviors at unloading tended to have higher blood levels of cortisol and CK.

## 5. Lairage

This section presents studies on the lairage phase and on the movement of the animals from the lairage pen to the stunning area. Lairage is regulated by the Council Regulation (EC) No 1099/2009 on the protection of animals at the time of the killing [35]. According to EFSA recommendations, in this phase the absence of hunger and thirst, adequate environment, thermal comfort, appropriate behavior, and freedom from pain and fear should be assessed [5].

### 5.1. Good Feeding during Lairage

Studies on water and feed provision during lairage were not found for heavy pigs. With respect to the need for water, the legislation [35] states that “water supply system in pens shall be designed, constructed and maintained so as to allow all animals at all times access to clean water without being injured or limited in their movements”. In Italy, 45% of the lairage pens inspected had a mean ratio of 1 drinker every 13 pigs [14].

### 5.2. Good Housing during Lairage

To the best of the author’s knowledge, only two studies considered lairage facilities in heavy pigs. The first one [14] recorded lairage conditions, reporting that during lairage pigs were housed at a stocking density of 0.63 m^2^/100 kg, lower than the EU legislation provision for the protection of pigs on farms (1.00 m^2^ per pig above 110 kg) [36]. According to the second study [19], the risk ratio of pigs DIP (Dead In Pen) was lower in the slaughterhouse which applied a lower stocking density (0.64 m^2^/100 kg vs. 0.56 and 0.46 m^2^/100 kg). Moreover, a lower DIP prevalence was associated with the building having large open windows on the roof and sidewalls, low brightness lights (40 lux), and high-pressure sprinklers as cooling devices.

### 5.3. Good Health during Lairage

The majority of studies on heavy pigs during lairage focused on the prevalence of skin lesions or traumas (as indicators of aggressive behaviors) in relation to lairage duration. The ABMs were mainly assessed after slaughter (i.e., on the carcasses), and association analysis was used to evaluate the effect of lairage duration on aggression outcomes.

Skin lesions can be assessed using different methods: as described in the Welfare Quality protocol [14,25] or using other scales [15,24]. The results differ between studies: some authors [25] did not find any association between skin lesions and overnight lairage, while others [15,24] observed increased lesions in the anterior part of the body after overnight lairage. It is important to consider that these three studies used different scoring systems and that they were carried out in different slaughterhouses and different years, therefore under different management practices, as well as lairage conditions, and this can explain the different results among studies.

A study [14] applying the Welfare Quality scoring system in four Italian slaughterhouses and in plants from other countries (Portugal, Finland, Brazil, and Spain), reported that skin lesion score was lower in Italian heavy pigs compared to other countries. The authors hypothesized that this may happen because in the PDO Parma Ham production mixing different batches, and when possible pens, is generally avoided, and lairage pens tend to be smaller (on average 19 pigs/pen) compared to other countries. Moreover, they reported that stocking density during lairage was similar to other countries, even considering the higher live weight of these pigs.

Ham defects have been proposed as post-mortem ABMs to assess animal welfare in the pre-slaughter phases. The assessment of ham defects may offer new insights on the welfare assessment of Italian heavy pigs since tight quality is routinely assessed on the day after slaughter to identify possible defects which may make the tights unsuitable for dry-curing. For example, muscle tears and hematomas identified at this stage might be caused by unloading, or be a consequence of fighting during lairage or poor handling [5]. Additionally, abscesses can have a traumatic or infective origin, and their development might be favored by many conditions such as the host immunity and physiology, stress, or the presence of other illnesses at the farm of origin.

The assessment of ham defects is always made by professionally-trained operators belonging to Parma Ham Consortium, following specific standards of quality [6]. The presence of defects determines the downgrading of the ham, or even its exclusion from the PDO circuit.

Studies available in the literature show conflicting results. On the one hand [25], increased abscess and muscle tear prevalence have been observed in hams from batches of pigs subjected to overnight lairage, suggesting that fighting might have led to increased slipping, and subsequent occurrence of muscle tears. According to the authors, overnight lairage may also exacerbate previous infective conditions. On the other hand [24], reduced petechial hemorrhaging and increased veining defects were reported in pigs subjected to overnight lairage, hypothesizing important physiological changes due to the interaction between prolonged stress and the increased time to rest before slaughter. Other studies found either an increase of veining defect after long lairage [26], or no association between overnight lairage and veining defect [25]. These studies were conducted in different slaughterhouses, therefore additional research should be carried out to allow a more complete understanding of lairage conditions (e.g., floor, illumination, air quality, stocking density, drinkers, presence of bedding and enrichment materials, human–animal relationship) and of the effect of overnight lairage on pig welfare and ham defects.

According to Gottardo et al. [23], overnight lairage increased the prevalence of esophageal-gastric ulceration (OGU) compared to slaughter on the day of arrival. The authors argued that prolonged fasting in pigs subjected to overnight lairage may have worsened the severity of OGUs.

Pigs subjected to overnight lairage showed a significantly higher percentage (6.4% vs. 5.1%) of batches with at least one carcass deemed to be unsuitable for consumption at the veterinary inspection compared to pigs slaughtered on the day of arrival [18]. It has also been reported that mortality during lairage (DIP) was mainly dependent on the slaughterhouse [19]. The season had also an effect, with the highest mortality being observed during the summer months (risk ratio = 1.14), peaking in July (risk ratio = 1.27). Additionally, THI affected DIP occurrence, with a significant breakpoint at 73.6 THI (corresponding to 30 °C and 20% RH, 26 °C and 50% RH, or 24 °C and 80% RH) [19]. The study associated specific lairage characteristics (high stocking densities, scarcity of windows, bright lights, and absence of sprinklers as cooling devices) to DIP, and found no relationship between length of transport and DIP [19].

The impact of stress during lairage has been also assessed in relation to its meat quality effects. Since pre-slaughter stress might affect meat quality characteristics (i.e., PSE or DFD (Dark, Firm, Dry) meat, pH, color, tenderness, water holding capacity, etc.), it might be used as a post-mortem indicator of pre-slaughter stressful conditions.

Overnight lairage was the main factor influencing meat quality in pigs with different halothane genotypes [22]. Overnight lairage led to a depletion of glycogen, inducing a decrease of pH1 (measured 90 min post mortem), color coordinates, and drip loss values, and an increase in ultimate pH (measured 24 h post mortem). Meat from pigs subjected to overnight lairage had a lower prevalence of the PSE defect (an indicator of short-term pre-slaughter stress) without showing increased levels of the DFD defect (an indicator of long-term pre-slaughter stress). Previous studies in lighter pigs showed that extended lairage reduces the proportion of PSE and increases the tendency towards DFD meat [37]. The difference could be imputable to differences in the age and weight of pigs. Older and heavier pigs indeed might have more glycogen resources in their muscles, therefore the DFD defect might occur with a different onset time.

## 6. Stunning

Stunning is the process that causes loss of consciousness in animals, and it is applied to reduce fear and pain. It is regulated by Council Regulation (EC) No 1099/2009 on the protection of animals at the time of slaughter or killing [35], and no specific provisions exist for the different categories of pigs in the EU regulation, nor in the PDOs production rules. This phase includes also moving pigs from the lairage to the stunning area, and pig restraining during stunning.

Depending on the technique used, stunning methods can be classified as electrical, mechanical, and gaseous [5]. In heavy pig production, the main methods used are electrical and CO_2_ stunning. According EFSA [5], the main negative welfare consequences of stunning can be pain, fear, and respiratory distress. Not many studies assessed the effect of stunning in Italian heavy pigs, and only two studies [14,27] (both reviewed in Section 6.1 below) were found with this respect. No studies were found on the welfare of heavy pigs when moved from lairage to stunning.

### 6.1. Good Health (Absence of Pain Induced by Management Procedures) at Stunning

The study from Nodari et al. [27] assessed the percentage of pigs correctly stunned after the application of electrical stunning in four Italian commercial abattoirs. In the study, three abattoirs used head-only manual tongs placed in two cases below the base of the ear (≥1.3 A) and one case between the eye and the ear (0.4–2 A). One abattoir used automatic head-to-chest stunning (≥2.5 A below the ear and ≥1.0 A in correspondence of the hearth). The study analyzed signs of consciousness to assess the welfare of the animals. An animal was defined as conscious if there was the presence of rhythmic breathing and/or pain reaction to nose prick and/or righting reflex and/or the animal appeared alert.

The authors reported that the position of the tongs was correct, none of the animals screamed (*n* = 1620), and no signs of skin burn or poor electrode maintenance was observed. Overall, 98.2% of animals were classified as unconscious, 1.1% were conscious and 0.7% were doubtful. Among the abattoirs, the one using head-to-chest stunning showed a lower rate of return of sensibility. The main additional signs observed in the case of return of consciousness were tongue movements (35%), vocalization (18%), corneal reflex (12%), and eyelid reflex (12%).

In the study by Dalmau et al. [14], stunning effectiveness was measured considering corneal reflex, righting reflex, rhythmic breathing, and vocalization, as indicated by EFSA in 2004 [38]. Overall, a large variability was observed among slaughterhouses in the return of consciousness and in the main ABMs. The study compared the assessments made in five countries using the methods proposed in the Welfare Quality protocol for the assessment of pig welfare at slaughter. The comparison in Italy was hard to make and further research should be carried out, because only two slaughterhouses used CO_2_, with very different results in both rhythmic breathing (approximatively 20 and 40% of prevalence) and corneal reflex (approximatively 0 and 30% of prevalence). The difference in corneal reflex was imputable to the fact that, with CO_2_ stunning, most pigs have closed eyes, and this may impair the evaluation of the corneal reflex, with the risk of overestimating it due to the confounding effect of the palpebral reflex. Recently, the use of CO_2_ for stunning pigs has been condemned worldwide due to severe criticism caused by the time elapsed before the loss of consciousness [39]. Interestingly, already in 2009, the European Regulation on the protection of animals at the time of killing [35] suggested that the use of carbon dioxide for pigs should be phased out in the future, but a recommendation was not made because abandoning this method was not deemed to be economically viable at the time the Regulation itself was enforced. Loss of consciousness can occur after 20 s of gas exposure, and during this period severe pain, fear, and behavioral distress have been observed [39], therefore electrical stunning, due to its faster effect, could be considered as a more valid alternative. This is likely to happen also in heavy pigs, therefore further studies on the effectiveness of this stunning methods and on the use of gas mixtures other than CO_2_ are recommended [40].

## 7. Conclusions

From this narrative review it is possible to conclude that the knowledge about the welfare of Italian heavy pigs in the pre-slaughter phase is scarce. Very little data exist, and the limited research available generally assesses meat and ham quality instead of relying on animal-based measures. The available data are partial and do not cover all the aspects or welfare principles during each of the pre-slaughter phases. Moreover, assessments have been mostly carried out in a single slaughterhouse, with a possible confounding effect due to abattoir personnel, practices, equipment, and management. Last but not least, the assessment of ABMs was often carried out using non-standardized methods, making comparisons among studies even more difficult. An epidemiological analysis covering all the principles in each of the pre-slaughter phases is recommended to enhance the welfare of this category of pigs, as well as the (ethical, qualitative, and commercial) value of their meat.

## Figures and Tables

**Table 1:** Main results of the literature research. The literature was divided according to the pre-slaughter phase. Animal-based measures (ABMs) were classified based on whether they were measured or collected before or after exsanguination.

Pre-Slaughter Phase	ABMs			
	Before Slaughter	After Slaughter	References	Method Taken From ^1^
Transport		Skin lesion	[11,12]	[13]
			[14]	[10]
	Body surface temperature		[11]	-
		Ham defects	[15]	[6]
		Stress hormones	[16,17]	-
	Deaths/losses		[18,19]	-
			[14]	[10]
	Ocular temperature		[11]	-
		Carcass damage	[11]	-
	Behavior at loading		[20]	-
	Behavior at unloading		[17,20,21]	-
		Meat quality	[12,21]	-
	Painting, huddling, coughing, sneezing		[14]	[10]
			
Lairage				
		DFD, PSE meat	[22]	-
		Gastric ulceration	[23]	-
		Ham defects	[24,25,26]	[6]
	Death		[19]	-
		Carcass destroyed	[18]	-
		Skin lesion	[24,25]	[10]
			[22]	[13]
		Tail biting	[25]	[10]
		Stress hormones	[17,20]	-
		Meat quality	[21]	-
Stunning		Vocalization	[14]	[10]
			[27]	-
		Absence of corneal reflex	[14]	[10]
			[27]	-
		Animal alert, head uplift, pain reaction, rhythmic respiration, blinking, body shivering, chronic convulsion, chewing, nystagmus, eyelid reflex, snout shivering, tongue movements	[27]	-

^1^ Previously published assessment methods used in the papers cited in the same row of the column “References”.

## References

[1] Clonan A., Wilson P., Swift J.A., Leibovici D.G., Holdsworth M. (2015). Red and processed meat consumption and purchasing behaviours and attitudes: Impacts for human health, animal welfare and environmental sustainability. Public Health Nutr..

[2] Di Pasquale J., Nannoni E., Del Duca I., Adinolfi F., Capitanio F., Sardi L., Vitali M., Martelli G. (2014). What foods are identified as animal friendly by Italian consumers?. Ital. J. Anim. Sci..

[3] Manyukhina Y. (2017). Consumer Food Ethics: Considerations of Vulnerability, Suffering, and Harm. J. Agric. Environ. Ethics.

[4] Lin-Schilstra L., Fischer A.R.H. (2020). Consumer Moral Dilemma in the Choice of Animal-Friendly Meat Products. Sustainability.

[5] EFSA (2020). Welfare of pigs at slaughter. EFSA J..

[6] Consortium for Parma Ham Prosciutto di Parma (Parma Ham) Protected Designation of Origin. https://www.prosciuttodiparma.com/wp-content/uploads/2019/07/Parma_Ham_Specifications_Disciplinare_Consolidato_Nov_13.pdf.

[7] Di Pasquale J., Nannoni E., Sardi L., Rubini G., Salvatore R., Bartoli L., Adinolfi F., Martelli G. (2019). Towards the abandonment of surgical castration in pigs: How is immunocastration perceived by Italian consumers?. Animals.

[8] Vitali M., Nannoni E., Sardi L., Martelli G. (2021). Knowledge and Perspectives on the Welfare of Italian Heavy Pigs on Farms. Animals.

[9] Wu F., Vierck K.R., DeRouchey J.M., O’Quinn T.G., Tokach M.D., Goodband R.D., Dritz S.S., Woodworth J.C. (2017). A review of heavy weight market pigs: Status of knowledge and future needs assessment. Transl. Anim. Sci..

[10] Welfare Quality® (2009). Assessment Protocol for Pigs.

[11] Arduini A., Redaelli V., Luzi F., Dall’Olio S., Pace V., Costa L.N. (2017). Relationship between deck level, body surface temperature and carcass damages in Italian heavy pigs after short journeys at different unloading environmental conditions. Animals.

[12] Nanni Costa L., Lo Fiego D.P., Dall’Olio S., Davoli R., Russo V. (1999). Influence of loading method and stocking density during transport on meat and dry-cured ham quality in pigs with different halothane genotypes. Meat Sci..

[13] Barton Gade P., Warriss P.D., Brown S.N., Lambooij B., Schuette A. (1996). Methods of improving pig welfare and meat quality by reducing stress and discomfort before slaughter-methods of assessing meat quality. Landbauforsch. Voelkenrode. Sonderh. New information on welfare and Meat Quality of Pigs as Related to Handling, Transport and Lairage Conditions.

[14] Dalmau A., Nande A., Vieira-Pinto M., Zamprogna S., Di Martino G., Ribas J.C.R., da Costa M.P., Halinen-Elemo K., Velarde A. (2016). Application of the Welfare Quality^®^ protocol in pig slaughterhouses of five countries. Livest. Sci..

[15] Arduini A., Redaelli V., Luzi F., Dall’Olio S., Pace V., Costa L.N. (2014). Effect of transport distance and season on some defects of fresh hams destined for DPO production. Animals.

[16] Bozzo G., Padalino B., Bonerba E., Barrasso R., Tufarelli V., Zappaterra M., Ceci E. (2020). Pilot Study of the Relationship between Deck Level and Journey Duration on Plasma Cortisol, Epinephrine and Norepinephrine Levels in Italian Heavy Pigs. Animals.

[17] Sardi L., Gastaldo A., Borciani M., Bertolini A., Musi V., Martelli G., Cavallini D., Rubini G., Nannoni E. (2020). Identification of possible pre-slaughter indicators to predict stress and meat quality: A study on heavy pigs. Animals.

[18] Nannoni E., Liuzzo G., Serraino A., Giacometti F., Martelli G., Sardi L., Vitali M., Romagnoli L., Moscardini E., Ostanello F. (2016). Evaluation of pre-slaughter losses of Italian heavy pigs. Anim. Prod. Sci..

[19] Vitali A., Lana E., Amadori M., Bernabucci U., Nardone A., Lacetera N. (2014). Analysis of factors associated with mortality of heavy slaughter pigs during transport and lairage. J. Anim. Sci..

[20] Calà P., Tassone F., Comellini M., Cristina Ielo M., Volpelli L., Dall’Olio S., Nanni Costa L. (2009). Effect of different pre-slaughter procedures on behavioural and blood parameters in pig. Ital. J. Anim. Sci..

[21] Sardi L., Gastaldo A., Borciani M., Bertolini A., Musi V., Garavaldi A., Martelli G., Cavallini D., Nannoni E. (2020). Pre-slaughter sources of fresh meat quality variation: The case of heavy pigs intended for protected designation of origin products. Animals.

[22] Nanni Costa L., Lo Fiego D.P., Dall’Olio S., Davoli R., Russo V. (2002). Combined effects of pre-slaughter treatments and lairage time on carcass and meat quality in pigs of different halothane genotype. Meat Sci..

[23] Gottardo F., Scollo A., Contiero B., Bottacini M., Mazzoni C., Edwards S.A. (2017). Prevalence and risk factors for gastric ulceration in pigs slaughtered at 170 kg. Animal.

[24] Bottacini M., Scollo A., Edwards S.A., Contiero B., Veloci M., Pace V., Gottardo F. (2018). Skin lesion monitoring at slaughter on heavy pigs (170 kg): Welfare indicators and ham defects. PLoS ONE.

[25] Vitali M., Bosi P., Santacroce E., Trevisi P. (2021). The multivariate approach identifies relationships between pre-slaughter factors, body lesions, ham defects and carcass traits in pigs. PLoS ONE.

[26] Nanni Costa L., Lo Fiego D.P., Tassone F., Russo V. (2005). Effect of Resting Time of Pigs and Pre-Chilling Time of Thighs on the Veining Defect of Parma Dry-Cured Ham. Vet. Res. Commun..

[27] Rota Nodari S., Polloni A., Giacomelli S., Vezzoli F., Galletti G. (2014). Assessing pig welfare at stunning in Northern Italy commercial abattoirs using electrical method. Large Anim. Rev..

[28] (2005). EC COUNCIL REGULATION (EC) No 1/2005 of 22 December 2004 on the protection of animals during transport and related operations and amending Directives 64/432/EEC and 93/119/EC and Regulation (EC) No 1255/97. Off. J. Eur. Union.

[29] (2020). MIPAAF (Ministero delle Politiche Agricole Alimentari e Forestali) Proposta di modifica del Disciplinare di produzione della denominaizone di origine protetta Prosciutto di Parma. Gazz. Uff..

[30] (2018). European Commission - Directorate-General for Health and Food Safety. Guide to Good Practices for the Transport of Pigs (2017-rev1 May 2018). https://op.europa.eu/en/publication-detail/-/publication/4909b6cd-7ec2-11ea-aea8-01aa75ed71a1.

[31] Gonzalez-Rivas P.A., Chauhan S.S., Ha M., Fegan N., Dunshea F.R., Warner R.D. (2020). Effects of heat stress on animal physiology, metabolism, and meat quality: A review. Meat Sci..

[32] Nanni Costa L. (2009). Short-term stress: The case of transport and slaughter. Ital. J. Anim. Sci..

[33] Brandt P., Aaslyng M.D. (2015). Welfare measurements of finishing pigs on the day of slaughter: A review. Meat Sci..

[34] Warner R.D., Kauffman R.G., Greaser M.L. (1997). Muscle protein changes post mortem in relation to pork quality traits. Meat Sci..

[35] (2009). EC COUNCIL REGULATION (EC) No 1099/2009 of 24 September 2009 on the protection of animals at the time of killing. Off. J. Eur. Union.

[36] (2008). EC COUNCIL DIRECTIVE 2008/120/EC of 18 December 2008 laying down minimum standards for the protection of pigs. Off. J. Eur. Union.

[37] Warriss P.D., Brown S.N., Edwards J.E., Knowles T.G. (1998). Effect of lairage time on levels of stress and meat quality in pigs. Anim. Sci..

[38] (2004). EFSA Opinion of the Scientific Panel on Animal Health and Welfare (AHAW) on a request from the Commission related to welfare aspects of the main systems of stunning and killing the main commercial species of animals. EFSA J..

[39] Mota-Rojas D., Bolanos-Lo D., Concepcion M., Ramirez-Te J., Roldan-San P., Flores-Pei S., Mora-Medin P. (2012). Stunning Swine with CO_2_ Gas: Controversies Related to Animal Welfare. Int. J. Pharmacol..

[40] Steiner A.R., Flammer S.A., Beausoleil N.J., Berg C., Bettschart-Wolfensberger R., Pinillos R.G., Golledge H.D.R., Marahrens M., Meyer R., Schnitzer T. (2019). Humanely Ending the Life of Animals: Research Priorities to Identify Alternatives to Carbon Dioxide. Animals.

